# Editorial: New Insights into the Pathophysiology and Treatment of Neonatal Hypoxic–Ischemic Encephalopathy

**DOI:** 10.3389/fneur.2016.00192

**Published:** 2016-11-01

**Authors:** Paulo Henrique Rosado-de-Castro, Rosalia Mendez-Otero, Pedro Moreno Pimentel-Coelho

**Affiliations:** ^1^Instituto de Ciências Biomédicas, Universidade Federal do Rio de Janeiro, Rio de Janeiro, Brazil; ^2^Departamento de Radiologia, Faculdade de Medicina, Universidade Federal do Rio de Janeiro, Rio de Janeiro, Brazil; ^3^Instituto de Biofísica Carlos Chagas Filho, Universidade Federal do Rio de Janeiro, Rio de Janeiro, Brazil

**Keywords:** neonatal hypoxic–ischemic encephalopathy, therapeutic hypothermia, preclinical models, translational research, clinical research

**The Editorial on the Research Topic**

**New Insights into the Pathophysiology and Treatment of Neonatal Hypoxic–Ischemic Encephalopathy**

Neonatal hypoxic–ischemic encephalopathy (HIE) is one of the leading causes of death and disability-adjusted life years (DALYs) in term infants (1, 2). This research topic presents a collection of one original article and four reviews covering translational research in HIE, including the utilization of animal models to unravel the pathophysiology of the disease, with a special focus on the development of novel therapies and the optimization of therapeutic hypothermia, the standard treatment for HIE.

In an elegant review, Davidson et al. addressed the utilization of therapeutic hypothermia for HIE. They discussed the evolution of hypoxic–ischemic brain damage and the optimal moment to start the hypothermic treatment, as well as the length and intensity of the intervention. The authors pointed out that, although hypothermia protocols are fairly close to optimal (starting within the first 6 h of life and continuing for 48–72 h), they are only partly effective, since the number needed to treat is currently of approximately eight patients. Therefore, they argued that additional enhancements may come from better detection of patients who could benefit from the therapy, and by determining the optimal rate of rewarming. Moreover, the authors examined the possibility of combining therapeutic hypothermia with other promising experimental therapies such as erythropoietin and cell transplantation.

In this regard, a mini-review by Lange discussed the possibility of combining peptidylarginine deiminases (PADs) inhibition with therapeutic hypothermia to improve its results. PADs are responsible for posttranslational deimination, which impact structural and functional properties of proteins. Consequently, PADs have a role in different developmental processes, and their dysfunction has been linked with many degenerative and inflammatory disorders. The author discussed the roles of PADs after a hypoxic–ischemic insult, including aspects such as epigenetics, phagoptosis, and autophagy, and commented on the possible therapeutic applications of selective or non-selective PAD inhibition for HIE.

The review article by Rumajogee et al. summarized the etiology and pathophysiology of cerebral palsy, and then described the Rice-Vannucci model and 12 other rodent models of HIE, detailing the characteristics, advantages, and limitations of each one of them. The authors mentioned important clinical improvements for HIE that were based on extensive studies in preclinical models, including therapeutic hypothermia and the importance of maintaining cerebral perfusion. Moreover, they discussed sex-related distinctions in response to perinatal injury as well as the main limitations of rodent models, including differences in brain structure and development between rodents and humans, which may have important implications for the development of new therapies. Finally, they compared rodent versus large animal models and intra- versus extra-uterine models.

In their review paper, Kang and Kadam have also pointed out the need for establishing preclinical models to study long-term effects and to develop novel therapies for neonatal seizures. The authors described diverse etiologies of neonatal seizures, including central nervous system infections and genetic diseases, while focusing on HIE. They provided a detailed discussion of the consequences of neonatal seizures on neurodevelopmental outcomes according to lesion type and severity, and called to the attention relevant aspects such as the evidence that prolonged seizures apparently lead to worsened brain injuries in HIE. The authors also reasoned that, because neonatal seizures are mostly subclinical, electroencephalographic monitoring and other parameters such as original lesion severity, antiepileptic drug efficacy, and follow-up magnetic resonance imaging are extremely important to estimate seizure burden and long-term outcomes.

This research topic also features an original article by Looney et al., who presented evidence that glial fibrillary acidic protein (GFAP) levels in umbilical cord blood serum samples do not differ among infants with perinatal asphyxia, infants with HIE, and healthy controls. Their data showed that GFAP levels were associated neither with the grade of HIE nor with the presence of developmental abnormalities at 36 months of age. Taken together with previous findings [Douglas-Escobar et al.; (3)], these results indicate that the release of the astrocytic intermediate filament GFAP into the blood is not an early event in HIE, although it has been shown that GFAP serum levels do increase at late time points, being associated with functional and morphological outcomes (4, 5). Therefore, the potential role of GFAP as a prognostic marker deserves further investigation, as well as the search for early predictive biomarkers that could identify patients who would benefit from therapeutic hypothermia within the first hour after birth.

In conclusion, the articles that comprise this research topic provide new insights into different aspects related to HIE and may be useful for researchers, and health-care students and professionals who want to stay updated.

## Author Contributions

PR-d-C, RM-O, and PP-C drafted and made substantial contributions to the conception of the work, approved the final version of the manuscript, and agreed to be accountable for all aspects of the work.

## Conflict of Interest Statement

The authors declare that the research was conducted in the absence of any commercial or financial relationships that could be construed as a potential conflict of interest.
